# Enhanced Photoluminescence and Random Lasing Emission in TiO_2_-Decorated FAPbBr_3_ Thin Films

**DOI:** 10.3390/nano13111761

**Published:** 2023-05-30

**Authors:** Xiaohong Liu, Caixia Xu, Hongquan Zhao

**Affiliations:** 1 Chongqing University, Shapingba, Chongqing 400044, China; 2 Chongqing Institute of Green and Intelligent Technology, Chinese Academy of Sciences, Chongqing 400714, China; 3 Chongqing School, University of Chinese Academy of Sciences, Chongqing 400714, China; 4 School of Primary Education, Chongqing Normal University, Chongqing 400700, China

**Keywords:** FAPbBr_3_, organic perovskite thin films, TiO_2_ nanoparticles, photoluminescence, random lasers

## Abstract

Herein, titanium-dioxide-decorated organic formamidinium lead bromide perovskite thin films grown by the one-step spin-coating method are studied. TiO_2_ nanoparticles are widespread in FAPbBr_3_ thin films, which changes the optical properties of the perovskite thin films effectively. Obvious reductions in the absorption and enhancements in the intensity of the photoluminescence spectra are observed. Over 6 nm, a blueshift of the photoluminescence emission peaks is observed due to 5.0 mg/mL TiO_2_ nanoparticle decoration in the thin films, which originates from the variation in the grain sizes of the perovskite thin films. Light intensity redistributions in perovskite thin films are measured by using a home-built confocal microscope, and the multiple scattering and weak localization of light are analyzed based on the scattering center of TiO_2_ nanoparticle clusters. Furthermore, random lasing emission with sharp emission peaks is achieved in the scattering perovskite thin films with a full width at the half maximum of 2.1 nm. The multiple scattering of light, the random reflection and reabsorption of light, and the coherent interaction of light within the TiO_2_ nanoparticle clusters play important roles in random lasing. This work could be used to improve the efficiency of photoluminescence and random lasing emissions, and it is promising in high-performance optoelectrical devices.

## 1. Introduction

Halide lead perovskite thin films have been witnessed to be one of the most promising candidates in the applications of light-emitting diodes [1,2,3], photovoltaic solar cells [4,5,6], high-resolution photodetectors and displays [7,8,9], low-threshold lasers [10,11,12], and optoelectrical devices [13,14,15], owing to their high conductivity [16], long carrier diffusion lengths [17], high defect tolerances [18,19,20], and good solution processabilities [21,22,23]. Among all these APbX_3_ perovskite thin films, methylammonium lead tribromide (MAPbBr_3_), formamidinium lead tribromide (FAPbBr_3_), and their mixtures have attracted great attention since the certified power conversion efficiency of solar cells improved from 3.8% (MAPbBr_3_) to 25.7% (MA_x_FA_1−x_PbBr_3_) within the past few years [24,25,26,27,28,29,30]. Meanwhile, the key parameters of perovskite devices, for example the photoluminescence quantum yield (PLQY), the photoresponsivity/detectivity and external quantum efficiency (EQE) of the photodetectors, and the thresholds of vertical-cavity surface-emitting lasers (VCSEL) and random lasers (RL), showed a development trend beyond expectations, which was ascribed to the advanced high-quality thin film growth techniques, interface engineering, surface texture processing, and electron and hole transport layer materials selection [31,32,33,34,35]. Recently, good linear and nonlinear optical properties were shown in FAPbBr_3_ thin films, and single-photon- and multiphoton-pumped micro/nanolasers and random lasers were all explored owing to their high gain, long carrier diffusion lengths, and good bandgap tuning ability [36,37,38,39,40]. To fulfill high-Q-factor and low-threshold micro- and nanolasers in FAPbBr_3_ thin films, ideal nanowires, triangular, square, hexagonal, or circular micro- and nanocrystals need to be grown or fabricated, and high-performance lasers could be realized by using the total internal reflection of the boundaries [41,42,43,44,45]. Unlike the former ones, random lasers in FAPbBr_3_ thin films do not require an internal reflection cavity, and they are generated by the random scattering of light, the coherent interaction of light, and the stimulated radiation light amplification in irregular disordered scattering media [46,47,48,49,50]. Along this line, Xu and coworkers realized two photon-pumped random lasers in FAPbBr_3_/polyethylene oxide (PEO)-composited thin films with good moisture and temperature stability, where PEO worked as a coagulant to form disordered islands, and as a spacer to isolate them from the humid air outside at the same time [51]. High-efficiency up-conversion random lasing from formamidinium lead bromide/amino-mediated silica sphere composites at room temperature with a high-quality (Q) factor of 1307 and a low threshold of 413.7 µJ cm^−2^ was studied by Tang and coworkers [52], which originated from the multiple random scattering of light and photon recycling of the silica spheres and the high optical gain of the perovskite quantum dots. Wang and coworkers obtained low-threshold green and red random lasing emission in inorganic halide lead perovskite microcrystals with plasmonic and interferential enhancement, in which gold and silver nanoparticles and patterned sapphire substrates were used to reduce the threshold of random lasers effectively [53]. Strong scattering structures, excellent gain media, and effective coherent resonance regions played key roles in improving the random lasing action performance in FAPbBr_3_ thin films [54,55].

Herein, enhanced photoluminescence and random lasing emission in titanium dioxide (TiO_2_)-decorated organic FAPbBr_3_ perovskite thin films grown by using the one-step spin-coating method were studied. An obvious reduction in absorption and over 8.5 nm blueshift of the photoluminescence emission were observed along with more TiO_2_ nanoparticle decoration in the thin films. The reabsorption and recycling of photons and grain size changes were attributed to the photoluminescence position shift. Multiple random scattering of light and weak localization were analyzed by using a home-built confocal microscope. Low-threshold random lasing emission with sharp emission peaks in the thin films were achieved in the scattering perovskite thin films. This work is promising in developing cavity-free nanolasers and optoelectrical devices.

## 2. Materials and Methods

**Figure 1:**
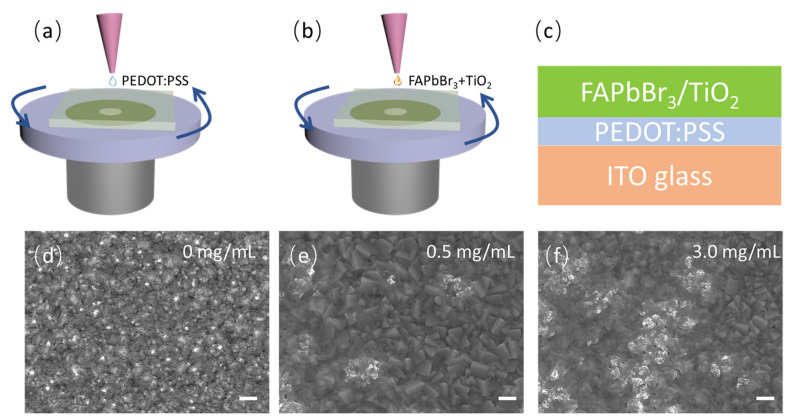
(**a**) Schematic of the growth of PEDOT:PSS buffer layer by using one-step spin-coating method; (**b**) schematic of the growth of FAPbBr_3_(TiO_2_) active layer by using one-step spin-coating method; (**c**) the structure of the prepared FAPbBr_3_/TiO_2_ thin films on ITO-coated glass; the surface appearance of (**d**) FAPbBr_3_ thin films; (**e**) 0.5 mg/mL and (**f**) 3.0 mg/mL TiO_2_-nanoparticle-decorated FAPbBr_3_ thin films measured by using a scanning electron microscope (the scale bar is 1 µm).

The precursor solutions of FAPbBr_3_ were anhydrous CH_5_N_2_Br bought from Sigma Aldrich (purity > 99.0%) and PbBr_2_ bought from 3A Materials (purity > 99.999%, Shanghai, China), which were dissolved in N,N-dimethylformamide (DMF, Alfa Aesar, purity > 99.8%, Shanghai, China) at a concentration ratio of 1.5:1, which was prepared in the glove box (water and oxygen component < 0.1 ppm). TiO_2_ rutile nanoparticle suspensions (Sigma-Aldrich, purity > 99.7%, average particle size around 25 nm, Shanghai, China) were added into the precursor solutions with concentrations of 0 mg/mL, 0.5 mg/mL, 1.0 mg/mL, 1.5 mg/mL, 2.0 mg/mL, 2.5 mg/mL, 3.0 mg/mL, 4.0 mg/mL, and 5.0 mg/mL. Then, 40 wt% FAPbBr_3_ solutions with TiO_2_ nanoparticle decoration in DMF were obtained after stirring the solution for more than 12 h by using a magnetic stirrer at room temperature. Before growing the perovskite thin films, ITO-coated glass substrates were cleaned by an ultrasonic cleaner where deionized water, acetone, ethanol, and then acetone were used, respectively. After that, the cleaned substrates were heated in a drying oven at 120 degrees to remove chemical residues and then treated with ultraviolet ozone for 15 min to improve the quality of the thin films grown by using solution-processed method. As seen in Figure 1a, Poly(3,4-ethylenedioxythiophene)/poly(styrenesulfonate) (PEDOT: PSS, 3A Materials, Shanghai, China) thin film was first coated on the cleaned ITO glass substrates by using the spin coater (4500 round/min, 60 s) to improve the morphology and quality of the active perovskite layers and annealed at 120 °C for 20 min. Finally, FAPbBr_3_ solutions with different concentrations of TiO_2_ nanoparticle decoration were grown by using the spin coater (2500 r/min, 60 s) with a thickness of around 60 nm, and the thin films were annealed at 80 °C for 20 min, as shown in Figure 1b. After completing the above operations, FAPbBr_3_ perovskite thin films with and without TiO_2_ nanoparticle decoration were prepared on the ITO glass substrate with the PEDOT:PSS buffer layer, as illustrated in Figure 1c.

## 3. Results and Discussion

The morphology of the FAPbBr_3_ perovskite thin films was analyzed by using a scanning electron microscope (SEM, JEOL JSM-7100F, Tokyo, Japan), as seen in Figure 1d,e. Without TiO_2_ nanoparticle decoration, the surface of the FAPbBr_3_ perovskite thin films is evenly and uniformly distributed. However, the surface of the FAPbBr_3_ perovskite thin films became more undulated, and the grain size became smaller with TiO_2_ nanoparticle decoration. The grain sizes might be important in the peak position and photoluminescence efficiency of the FAPbBr_3_ thin films. TiO_2_ nanoparticles disperse onto the surface of the film, and randomly distributed scattering centers are formed, spreading across the whole thin film. As the concentration of TiO_2_ increases, more scattering centers are obtained, which is conducive to improving the luminescence efficiency of the material and realizing low-threshold random lasing.

**Figure 2:**
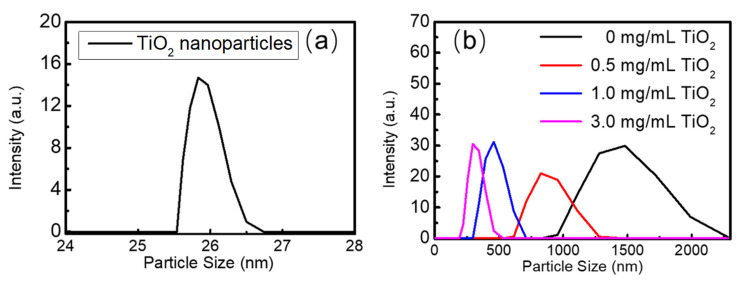
(**a**) The particle size distribution of TiO_2_ nanoparticles; (**b**) the particle size distribution of FAPbBr_3_ crystals with concentrations of TiO_2_ at 0 mg/mL, 0.5 mg/mL, 1.0 mg/mL, and 3.0 mg/mL.

The granularity of the TiO_2_ nanoparticles and the perovskite thin films were analyzed by using a particle size analyzer (Zetasizer Nano ZS90, Malvern Panalytical Ltd., Malvern, United Kingdom). As seen in Figure 2a, the particle sizes of TiO_2_ nanoparticles vary from 25 nm to 27 nm, which are close to the parameters from the raw material supplier. As seen in 2b, the particle size distribution of the FAPbBr_3_ perovskite grains changes from 1480 nm to 294 nm, and the number of grains decreases with the increasing concentration of TiO_2_ nanoparticles. The optical properties of the FAPbBr_3_ perovskite thin films may be affected by the scattering of the grain boundaries, the reabsorption and recycling of light, and multiple scattering of light along with the varied grain sizes, which might be used to modulate the photoluminescence peak position and random lasing modes and thresholds in the FAPbBr_3_ perovskite thin films using TiO_2_ nanoparticle decoration.

**Figure 3:**
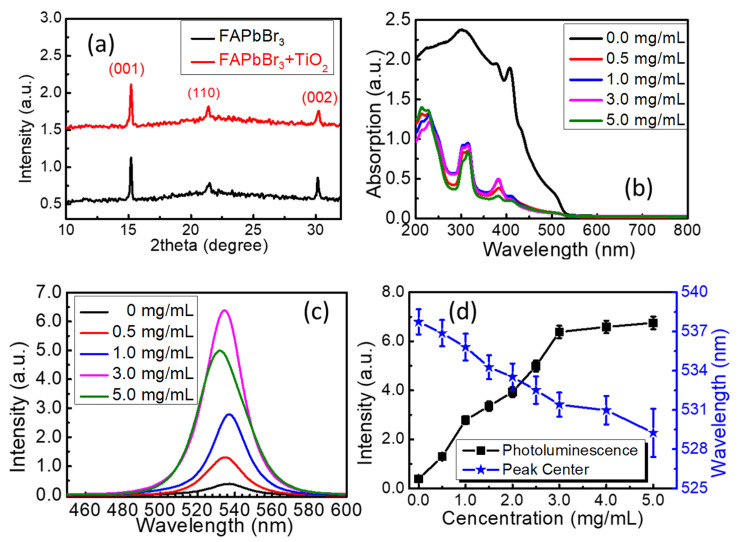
(**a**) X-ray diffraction spectra of the FAPbBr_3_ and FAPbBr_3_/TiO_2_ thin films; (**b**) room-temperature ground-state absorption spectra of the FAPbBr_3_ and FAPbBr_3_/TiO_2_ thin films; (**c**) photoluminescence emission spectra of the FAPbBr_3_ and FAPbBr_3_/TiO_2_ thin films excited by a continuous-wave laser at 405 nm; (**d**) the photoluminescence emission intensity and wavelength versus the concentration of TiO_2_ nanoparticles.

The unit structure of the FAPbBr_3_/TiO_2_ thin films was characterized by using an X-ray diffractometer (D/MAX-rB, Rigaku, Japan). As seen in Figure 3a, three prominent diffraction peaks located at 15.22°, 21.40°, and 30.26° could be seen in the X-ray diffraction spectra, which correspond to the (001), (110), and (002) peaks of the typical ABX_3_ perovskite structure that was determined by using the database in the JCPSD database. The room-temperature absorption spectra were measured using a UV-VIS-NIR spectrometer (Shimadzu, UV-2600, Guangzhou, China), as shown in Figure 3b. The bandgaps of the FAPbBr_3_ and FAPbBr_3_/TiO_2_ thin films were calculated by using the Tauc-plotted curves of the absorption spectra as 2.313 eV, 2.322 eV, 2.325 eV, 2.329 eV, and 2.330 eV, respectively, which related to the hybrid Br 4p and Pb 6s orbitals on the valence band and the Pb 6p orbitals on the conduction bands [56]. A noticeable reduction in the absorption spectra is observed above the bandgap. The photoluminescence was excited by a continuous-wave laser centered at 405 nm that was focused using a lens with a diameter of 1.0 mm, and the pumping power of the laser was set as 100 mW. The photoluminescence emission spectra were collected using an optical fiber that was connected to a spectrometer (Ocean Optics Inc., QE65Pro, Shanghai, China). As shown in Figure 3c, the photoluminescence emission spectra around 530 nm could be observed, which originate from the PbBr_6_ octahedra in the ABX_3_ perovskite structure, and are the typical spontaneous emission from the exciton recombination process [57]. The photographs of the FAPbBr_3_ thin films with and without TiO_2_ nanoparticle decoration were taken using a high-resolution camera, as seen in Figure S1 of the Supplementary Information (SI). The green photoluminescence in the FAPbBr_3_ without TiO_2_ nanoparticle decoration is relatively weak; however, it increased dramatically along with the concentration of TiO_2_ nanoparticles. As exhibited in Figure 3d, the intensity of photoluminescence emission increased along with the concentration of TiO_2_ nanoparticles until it reached 3.0 mg/mL. As high as a 16-fold enhancement of the photoluminescence emission was achieved with 3.0 mg/mL TiO_2_ nanoparticle decoration compared with that of the pristine FAPbBr_3_ membrane. A further increase in the concentration of TiO_2_ lead to a decrease in the fluorescent intensities, together with an 8.5 nm blueshift of the fluorescent peak. In addition, an over 8.5 nm blueshift of the photoluminescence emission peak was measured when the concentration of TiO_2_ nanoparticles reached 5.0 mg/mL. A gradual variation trend from 537.7 nm to 529.2 nm was observed when higher concentrations of TiO_2_ nanoparticles were decorated. To reduce the experimental errors and improve the reliability of the results from the optical measurement and the fiber spectrometer, the photoluminescence emission spectra of all the samples were recorded three times. The error bars for the photoluminescence intensity and peak centers were added, as shown in Figure 3d. The photoluminescence peak position could be attributed to different factors, such as the reabsorption and recycling of light, the temperature of the thin films, the grain size of the perovskite crystals, reabsorption combined with carrier diffusion, multiple scattering of light from disordered grain boundaries, and scattering centers of the TiO_2_ nanoparticle clusters [58]. In this work, the granularity changes in the FAPbBr_3_ perovskite grains play an essential role in the photoluminescence emission intensities and the emission peak centers [59]. In addition, the multiple scattering of light from the TiO_2_ nanoparticle clusters increases the light path thickness within the perovskite thin films, where the reabsorption and recycling of light and multiple reflections could occur. Moreover, the temperature accumulation in the thin films might also contribute to the blueshift of the photoluminescence position [60], since the temperature accumulation will intensify with more TiO_2_ nanoparticle clusters in the thin films.

**Figure 4:**
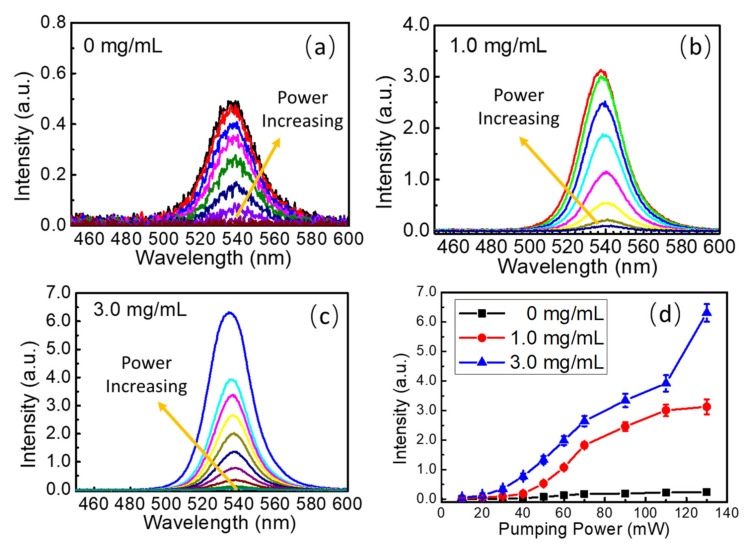
(**a**) Photoluminescence spectra of the FAPbBr_3_ thin films under different pumping power; (**b**) photoluminescence spectra of the FAPbBr_3_ thin films with 1.0 mg/mL TiO_2_ nanoparticle decoration under different pumping power; (**c**) photoluminescence spectra of the FAPbBr_3_ thin films with 3.0 mg/mL TiO_2_ nanoparticle decoration under different pumping power; (**d**) photoluminescence intensity changes in FAPbBr_3_ thin films with and without TiO_2_ nanoparticle decoration versus pumping power.

To explore in-depth the photoluminescence performance of the FAPbBr_3_ and FAPbBr_3_/TiO_2_ thin films, excitation-power-dependent photoluminescence spectra were measured at the range of 10 mW to 130 mW with an interval of 10 mW or 20 mW, as shown in Figure 4a–c. To provide repeatable experimental results, the beam size of the excitation laser source was set to 1.0 mm in diameter, which covers hundreds of TiO_2_ nanoparticle clusters. The photoluminescence emissions with different concentrations of TiO_2_ nanoparticle decoration were collected from the whole covered areas, and each sample was measured three times. The error bars were added to illustrate the experimental differences, as shown in Figure 4d, and a good repeatability of the photoluminescence is presented. The photoluminescent intensities of FAPbBr_3_ thin films with and without TiO_2_ decoration change as the pumping powers increase, as shown in Figure 4d. The intensity of the photoluminescence emission starts to increase under the power of 30 mW in the FAPbBr_3_ thin films, and it reaches the maximum under the illumination power of 90 mW as the absorption is saturated by the perovskite thin films. A similar process could be observed in FAPbBr_3_ thin films with 1.0 mg/mL TiO_2_ decoration. Still, the photoluminescent intensity increases under the illumination power of 10 mW, and the absorption saturation is reached at 110 mW. The photoluminescence intensity of the latter is almost 10 times larger than the former. In sharp contrast, the photoluminescence intensity in FAPbBr_3_ thin films with 3.0 mg/mL TiO_2_ decoration exhibits another high enhancement under the pumping power of 110 mW, which is nearly 16-fold compared with that of the pristine FAPbBr_3_ thin films. The multiple random scattering of light caused by the TiO_2_ nanoparticle clusters increases the reflection possibility of light and the adequate optical thickness of the perovskite thin films, and the reabsorption and recycling of light also affect the photoluminescence efficiency and peak position. All these factors make the photoluminescence efficiency increase dramatically along with the decoration concentration of TiO_2_ nanoparticles.

**Figure 5:**
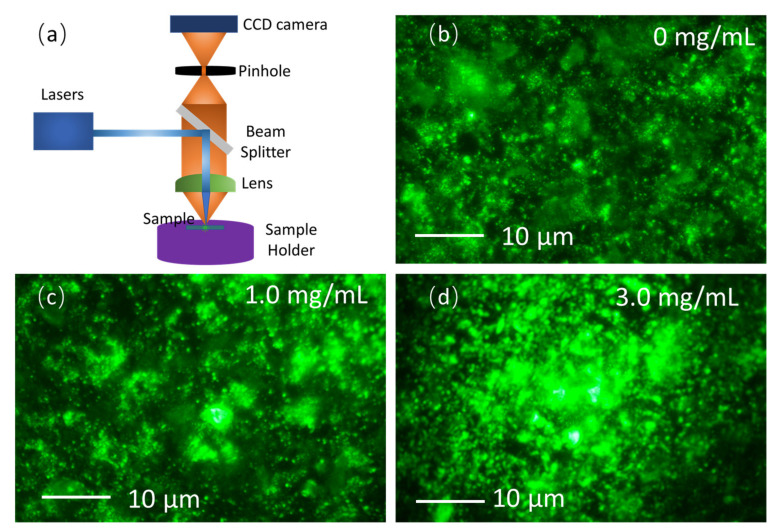
(**a**) Schematic of the home-built confocal microscope; micro-scale photoluminescence distribution in (**b**) FAPbBr_3_ thin films, (**c**) FAPbBr_3_ thin films with 1.0 mg/mL TiO_2_ nanoparticle decoration, and (**d**) FAPbBr_3_ thin films with 3.0 mg/mL TiO_2_ nanoparticle decoration.

To further study the physics behind the photoluminescence enhancement, a home-built confocal microscope was used to investigate the photoluminescence distribution at the micro-scale. As shown in Figure 5a, the white light source is collimated into a parallel beam by a lens system and passed through the beam splitter, and then the light source passes through the focusing objective lens to reach the sample surface. At the same time, another laser beam centered at 405 nm is focused on the same position through the reflection of the beam splitter to excite the fluorescence emission in the perovskite thin films. The photoluminescence emission emitted from the surface of the material returns through the reverse path to the CCD detector for imaging. As shown in Figure 5b, uniform photoluminescence with some bright spots on the grain boundaries could be observed in FAPbBr_3_ thin films. Figure 5c shows larger bright regions originating from multiple scatterings of light at the TiO_2_ nanoparticle clusters. Larger bright areas appear as the concentration of TiO_2_ nanoparticle is increased, as can be seen in Figure 5d. Photoluminescence distribution photographs of the FAPbBr_3_ thin films with concentrations of TiO_2_ nanoparticle decorations of 0 mg/mL, 0.5 mg/mL, 1.0 mg/mL, 1.5 mg/mL, 3.0 mg/mL, and 5.0 mg/mL were captured at a wide range using a low-magnification objective lens on the home-built fluorescence microscope, as seen in Figure S2 in SI. The photographs show the enhancement of the photoluminescence caused by the randomly distributed scattering centers and localized regions of light cross the whole thin films, and the enhancement of the photoluminescence also spreads across the whole of the thin films. Based on the photographs, the brighter photoluminescence regions originate from multiple scatterings of light and reabsorptions and recycling of light, and the photons are localized in some closed regions, which play important roles in the formation of random lasing emission, the weak localization of light, and the coherent resonance of light in the scattering media [53].

**Figure 6:**
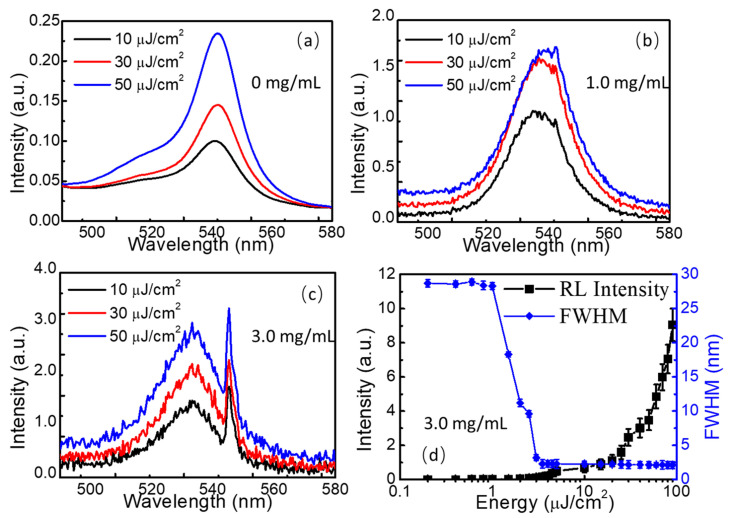
(**a**) Photoluminescence spectra of the FAPbBr_3_ thin films; (**b**) weak narrow peaks from random lasing emission in FAPbBr_3_ thin films with 1.0 mg/mL TiO_2_ nanoparticle decoration; (**c**) sharp random lasing emission spectra in FAPbBr_3_ thin films with 3.0 mg/mL TiO_2_ nanoparticle decoration; (**d**) the intensity of the output random lasing emission and FWHM changed along with the increase in excitation energy.

The scattering centers in Figure 5c,d are beneficial to the formation of cavity-free random lasers in the disordered media, where multiple scatterings and random reflections of light could take place within the FAPbBr_3_ thin films with TiO_2_ nanoparticle decoration. To study the random lasing action in TiO_2_-nanoparticle-decorated FAPbBr_3_ thin films, a nanosecond laser centered at 354.7 nm with the pulse frequency of 1.0 kHz and the pulse width of 5.0 ns was used as the excitation source and focused on the surface of the perovskite thin films with a spot diameter of 1.0 mm to cover hundreds of TiO_2_ nanoparticle cluster regions. As seen in Figure 6a, a broadband photoluminescence peak centered at 541.7 nm could be observed in FAPbBr_3_ thin films with a full width at half maximum (FWHM) of 12.8 nm, which is much narrower than that of the photoluminescence spectrum (24.2 nm). However, no random lasing emission was investigated in this thin film. With 1.0 mg/mL TiO_2_ nanoparticle decoration, some weak narrow peaks emerged at the right side of the photoluminescence spectrum, as seen in Figure 6b, which is the typical sign of random lasing emission. Only some stunted narrow peaks could be observed when the excitation energy surpassed 10 µJ/cm^2^. To improve the performance of random lasing action, sharp emission peaks with an FWHM of 2.1 nm were investigated in FAPbBr_3_ thin films with 3.0 mg/mL TiO_2_ nanoparticle decoration, as shown in Figure 6c. The intensity of the output random lasing emission and FWHM were recorded, as drawn in Figure 6d. Below the threshold, only broadband photoluminescence could be observed with an FWHM of 28 nm, which is even wider than the photoluminescence emission in Figure 6a. Above the threshold of around 5.0 µJ/cm^2^, the FWHM reduced to 2.1 nm rapidly, and the intensity of the sharp lasing emission increased dramatically with the excitation energy. As shown in Figure 4c,d, multiple scatterings and reflections of light from the photoluminescence occur due to the random distributed scattering centers formed by the TiO_2_ nanoparticle clusters and the grain boundaries of the FAPbBr_3_ crystals contributed to the formation of the random lasing emission. The photons are trapped in some closed regions, and the resonant interaction of light within the closed areas works as the feedback cavity of the F-P cavity lasers, and the stimulated emission of light could be occur from the scattering region when the gain surpasses the scattering and absorption loss of light. The high gain property of the FAPbBr_3_ perovskite thin films also plays a vital role in forming the random lasers. That is why random lasers are easier to observe in the FAPbBr_3_ thin films with more TiO_2_ nanoparticle decoration. As seen in Figure 6c, some other small peaks of random lasing emission could also be observed across the whole photoluminescence emission spectrum. These peaks originate from different localized regions of light, where the optical path is changed due to the random scattering and reflection of light across the TiO_2_ clusters in Figure 4d, and the reabsorption and recycling of light may also change the transport direction of light. The size of the closed region works as the cavity length of the traditional lasers, which could be used to modulate the laser modes.

The experimental results in this work provide a low-cost and easy-to-process method to enhance the photoluminescence emission and modulate the peak position of the FAPbBr_3_ thin films The nano- and microscale cavity-free low-threshold random lasers with an FHWM of 2.1 nm are promising for developing high-resolution light sources and displays, temperature and moisture sensors, and novel optoelectrical devices.

## 4. Conclusions

In conclusion, FAPbBr_3_ thin films without and with TiO_2_ nanoparticle decoration were grown by using the one-step spin-coating method. The granularity of the TiO_2_ nanoparticles and the perovskite thin films were analyzed by using a particle size analyzer with the particle sizes of TiO_2_ nanoparticles varying from 25 nm to 27 nm, and the particle size distribution of the FAPbBr_3_ perovskite grains changed from 1480 nm to 294 nm. An over 16-fold photoluminescence enhancement and a more than 8.5 nm blueshift of the photoluminescence emission peaks were measured, which originated from the grain size differences, the reabsorption and recycling of light, the thermal effect, and the scattering of light within the TiO_2_ nanoparticle clusters. The photoluminescence distribution photographs taken by the home-built confocal microscope verified the scattering contribution of the TiO_2_ nanoparticle clusters. Sharp random lasing emission spectra with the FHWM of 2.1 nm were detected with a low threshold of 1.0 µJ/cm^2^, where multiple scatterings and reflections of light, the resonant interaction of light, and the stimulated amplification of light are attributed to the formation of the random lasers. Some weak laser modes were observed that correspond to different sizes of the localized regions. This work provided a low-cost and easy-to-process method to improve the photoluminescence efficiency and achieve high-performance and low-threshold random lasers, which showed excellent application prospects in developing new light sources and high-resolution displays, sensors, and high-performance optoelectronic devices.

## Data Availability

Not applicable.

## Supplementary Materials

The following supporting information can be downloaded at https://www.mdpi.com/article/10.3390/nano13111761/s1, Figure S1: The photographs of the FAPbBr_3_ thin films with and without TiO_2_ nanoparticles decoration. Figure S2. The distribution of photoluminescence emission in the FAPbBr_3_ thin films with and without TiO_2_ nanoparticles decoration measured by using the home-built fluorescence microscope.
