# Supplementary material for: Enhanced Photoluminescence and Random Lasing Emission in TiO2-Decorated FAPbBr3 Thin Films
Source: Nanomaterials (Basel). 2023 May 30;13(11):1761. doi: 10.3390/nano13111761 (PMC10254827; doi:10.3390/nano13111761)
Supplement: Supplementary file 1 [file nanomaterials-13-01761-s001.zip › nanomaterials-2399443-supplementary.pdf]

Supplementary Information

Enhanced photoluminescence and random lasing emission in TiO_2-decorated $FAPbBr_3$ thin films

Xiaohong Liu[1,2,3], Caixia Xu[4,*], Hongquan Zhao[1,2,3,*]

[1] Chongqing University, Shapingba, Chongqing 400044, China; lxhsuda@163.com
[2] Chongqing Institute of Green and Intelligent Technology, Chinese Academy of Sciences, Chongqing 401120, China：hqzhao@cigit.ac.cn
[3] Chongqing School, University of Chinese Academy of Sciences, Chongqing 400714, China; hqzhao@cigit.ac.cn
[4] School of Primary Education, Chongqing Normal University, Chongqing, 400700 China
* Correspondence: noendness@126.com (C. Xu); hqzhao@cigit.ac.cn (H. Zhao).

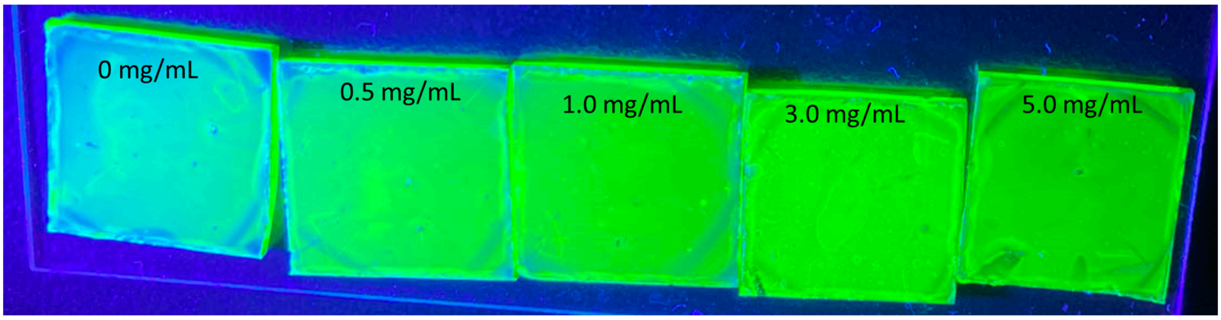

Figure S1 The photographs of the $FAPbBr_3$ thin films with and without TiO_2 nanoparticles decoration.

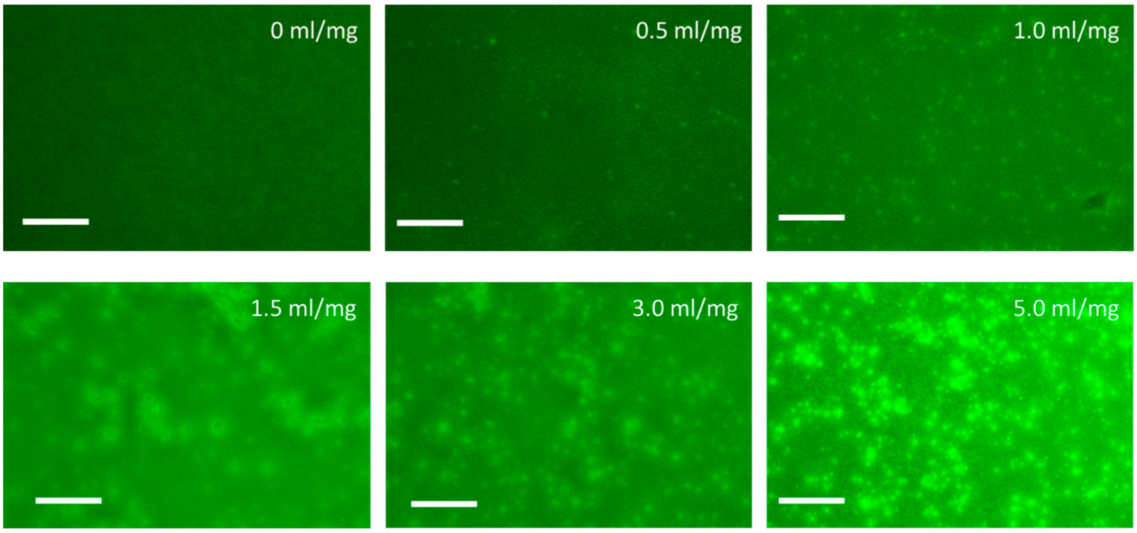

Figure S2 The distribution of photoluminescence emission in the $FAPbBr_3$ thin films with and without TiO_2 nanoparticles decoration measured by using the home-built fluorescence microscope (the scale bar is 100 μm).
